# P-7. A study of influenza vaccination response in disabled stroke patients

**DOI:** 10.1093/ofid/ofae631.218

**Published:** 2025-01-29

**Authors:** Achiraya Pakngao, Sureerat Suwatcharangoon, Pintip Ngamjanyaporn, Kobporn Boonnak, Tanitta Suangtamai, Porpon Rotjanapan

**Affiliations:** Faculty of Medicine Ramathibodi Hospital, Mahidol University, Bangkok, Krung Thep, Thailand; Faculty of Medicine Ramathibodi Hospital, Mahidol University, Bangkok, Krung Thep, Thailand; Faculty of Medicine Ramathibodi Hospital, Mahidol University, Bangkok, Krung Thep, Thailand; Faculty of Medicine Siriraj Hospital, Mahidol University, Bangkok, Krung Thep, Thailand; Faculty of Medicine Ramathibodi Hospital, Mahidol University, Bangkok, Krung Thep, Thailand; Faculty of Medicine Ramathibodi Hospital, Mahidol University, Bangkok, Krung Thep, Thailand

## Abstract

**Background:**

To date, no clinical study has confirmed the clinical efficacy of routine annual vaccination in stroke patients. The primary objective was to study the immunologic response to influenza vaccination between functional-dependence (FD) and functional-independence (FI) ischemic stroke patients.

Median hemagglutination titers at T0 and T1

**Figure:**
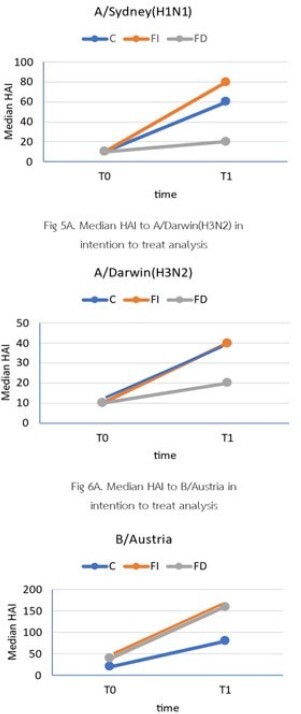

**Methods:**

A prospective observational study was conducted between 2023 and 2024 at Ramathibodi Hospital. Old ischemic stroke patients were screened for eligibility. Enrolled patients received standard doses of inactivated trivalent influenza vaccine. Data on patient demographics were retrieved. Blood samples were collected for hemagglutination inhibition (HAI) before and 4-8 weeks after vaccination to determine seroprotection and seroconversion rates.

**Results:**

As per the interim analysis, 198 patients were enrolled, and 161 individuals have completed the HAI analysis. The mean ages (±SD) of the control (C), FI, and FD groups were 69.4±7.4 years, 71.4±8.6, and 75.9±8.3, respectively (p< 0.001). The FD group had significantly higher baseline IFN-α2, IFN-γ, IL-6, IL-12p, and IL-18 levels than the FI and C groups.

Before (T0) and after (T1) vaccination, the proportion of participants with HAI titers >1:40 on the A/Sydney (H1N1) strain in the FI group was significantly higher than in the FD group (p=0.031 and 0.003 at T0 and T1, respectively) similarly to the A/Darwin (H3/N2) and the B/Austria strains and individuals achieved titer >1:160 (p=0.046 at T1). The C group displayed the highest seroconversion rates compared to the FI and the FD groups at 34.2%, 25.4%, and 19.4% (p=0.033). There were three probable and 23 possible influenza infections three months after enrollment. Twenty-two of 198 patients developed mild adverse events associated with influenza vaccination.

**Conclusion:**

The FI group achieved higher seroprotection and seroconversion rates than the FD group. The possible associated factors were higher levels of pro-inflammatory cytokines at baseline, resulting in better vaccination responses in the FI group. Therefore, the FD group may require a different influenza vaccination strategy to achieve more appropriate immune responses.

**Disclosures:**

**All Authors**: No reported disclosures

